# Environmental DNA sequencing primers for eutardigrades and bdelloid rotifers

**DOI:** 10.1186/1472-6785-9-25

**Published:** 2009-12-11

**Authors:** Michael S Robeson, Elizabeth K Costello, Kristen R Freeman, Jeremy Whiting, Byron Adams, Andrew P Martin, Steve K Schmidt

**Affiliations:** 1 University of Colorado, Department of Ecology and Evolutionary Biology, Ramaley N122, Campus Box 334, Boulder, CO 80309-0334, USA; 2 University of Colorado, Department of Chemistry and Biochemistry, 215 UCB, Boulder, CO 80309-0334, USA; 3 Brigham Young University, Department Biology and Evolutionary Ecology Laboratories, 775 WIDB, Provo, UT 84602-5253 USA

## Abstract

**Background:**

The time it takes to isolate individuals from environmental samples and then extract DNA from each individual is one of the problems with generating molecular data from meiofauna such as eutardigrades and bdelloid rotifers. The lack of consistent morphological information and the extreme abundance of these classes makes morphological identification of rare, or even common cryptic taxa a large and unwieldy task. This limits the ability to perform large-scale surveys of the diversity of these organisms.

Here we demonstrate a culture-independent molecular survey approach that enables the generation of large amounts of eutardigrade and bdelloid rotifer sequence data directly from soil. Our PCR primers, specific to the 18s small-subunit rRNA gene, were developed for both eutardigrades and bdelloid rotifers.

**Results:**

The developed primers successfully amplified DNA of their target organism from various soil DNA extracts. This was confirmed by both the BLAST similarity searches and phylogenetic analyses. Tardigrades showed much better phylogenetic resolution than bdelloids. Both groups of organisms exhibited varying levels of endemism.

**Conclusion:**

The development of clade-specific primers for characterizing eutardigrades and bdelloid rotifers from environmental samples should greatly increase our ability to characterize the composition of these taxa in environmental samples. Environmental sequencing as shown here differs from other molecular survey methods in that there is no need to pre-isolate the organisms of interest from soil in order to amplify their DNA. The DNA sequences obtained from methods that do not require culturing can be identified post-hoc and placed phylogenetically as additional closely related sequences are obtained from morphologically identified conspecifics. Our non-cultured environmental sequence based approach will be able to provide a rapid and large-scale screening of the presence, absence and diversity of Bdelloidea and Eutardigrada in a variety of soils.

## Background

Micro-invertebrates, though very important to the soil biocenose (self-regulating ecological communities) and energy flux of a system, are still poorly understood in terms of their taxonomy and geographical distributions [1-4]. Like many microfaunal organisms, Rotifera and Tardigrada pose problems for taxonomists and evolutionary biologists due to the difficulties associated with isolation, identification and enumeration of organisms that do not preserve any discernable morphological characters. Even when it is possible to successfully culture these organisms, limited phenotypic differentiation among taxa and cyclomorphosis (seasonal change in body shape; [5]) confound accurate taxonomy. This lack of consistent morphological information and the extreme abundance of meiofaunal organisms makes identification of rare, or even common, cryptic taxa a large and unwieldy task [6,4] as only painstaking microscopy can be used to identify synapomorphies.

Environmental sequencing is valuable for performing large-scale surveys of the diversity of organisms that cannot be cultured or grown in the laboratory or when species are difficult to distinguish using phenotypic characters. These issues argue for culture independent molecular surveys of meiofaunal diversity in natural ecosystems. Microbiologists have faced many of the same problems and solved them by turning to conserved DNA sequences as a means of describing communities [7,8]. Instead of isolating and culturing individuals, communities are characterized by extracting all of the DNA in a particular sample (soil, water, air), amplifying a specific gene using PCR, cloning individual PCR products, and then sequencing individual clones. This environmental DNA approach has revolutionized microbiology. For example, these techniques have been successfully used to provide new insights into fungi [9,10], novel Chloroflexi [11], abundance and distribution of *Psychrobacter *and *Exiguobacterium *[12] and have been used to provide information about the structure and function of alpine and arctic soil microbial communities [13].

Our survey focuses on the 18S rRNA gene, commonly used for phylogenetic inference of eukaryotes due to its highly conserved sequence and ability to resolve relatively deep nodes. This is the first description of the general utility of environmental DNA sequencing approaches for characterizing difficult to study ecological communities of eutardigrades and bdelloid rotifers.

Environmental sequencing as described here differs from other molecular survey methods [6,14] in that there is no need to pre-isolate the bdelloid rotifers or eutardigrades of interest from soil (or other mediums) before amplifying their DNA. The successful development of clade-specific 18s SSU primers has shown to be effective when surveying the diversity of targeted groups of organisms. For example, clade specific 18s SSU primers have been used to describe soil metazoans[15,16] and reveal the hidden diversity and biogeographic endemism of kinetoplastids (flagellate protozoa) [17].

The use of 18S rDNA allows for sequences to be combined into already existing 18S and 16S rDNA databases, including those being developed by microbial ecologists through their large scale molecular surveys as referred to above. Here we describe the utility of screening for bdelloid rotifer and eutardigrade diversity in two very distinct sample sites with targeted 18S primers: the high-elevation sites located within the Niwot Ridge Long Term Ecological Research (LTER) site in the Colorado Rockies, and the low-elevation sites located within the Calhoun Experimental Forest in South Carolina.

## Results

**Figure 1:**
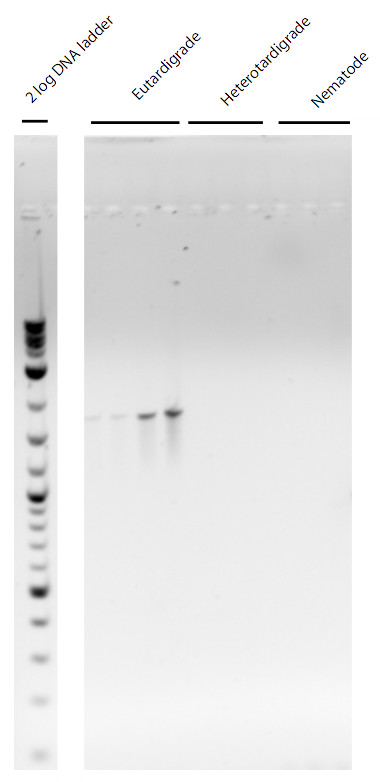
**Gel image of PCR results for eutardigrade specific 18s rDNA primers**. First four lanes are from replicate individuals from a single population eutardigrades. Lanes five through 7 are from heterotardigrades. Lanes eight through ten are nematodes.

**Figure 2:**
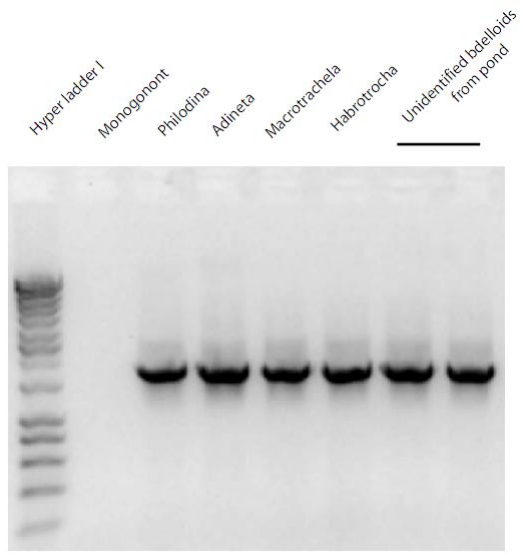
**Gel image of PCR results for bdelloid specific 18s rDNA primers**. Lane 1 is the Hyper ladder 1 from Bioline USA Inc. MA, *Brachionus plicatilis *is the Monogonont in lane 2. Lanes three through six are from individual representatives of the following bdelloid rotifers: *Philodina*, *Adineta*, *Macrotrachela*, and *Habrotrocha*. The final two lanes are from unidentified bdelloids taken from a pond.

We developed two forward primers for taxon specific amplification of eutardigrades and bdelloid rotifers. These primers were used in combination with a universal reverse 18S rDNA primer to specifically characterize the diversity of these two groups from several environments. PCR, BLAST and phylogenetic analysis confirmed that each set of primers amplifies the targeted groups with fidelity and specificity (Figures 1 &2). We have observed many invertebrates within the soils prior to DNA extraction and amplification, including mites, nematodes, and insects; none of these were observed within the sequencing data produced using the specific primers in this study. Thus, our primers are shown to be specific to the targeted groups of organisms. The closest known sequences or clades to the environmental sequences are noted below. Note that we do not infer that the environmental sequences are of the same species or genera to those closest to them.

### Tardigrada

**Figure 3:**
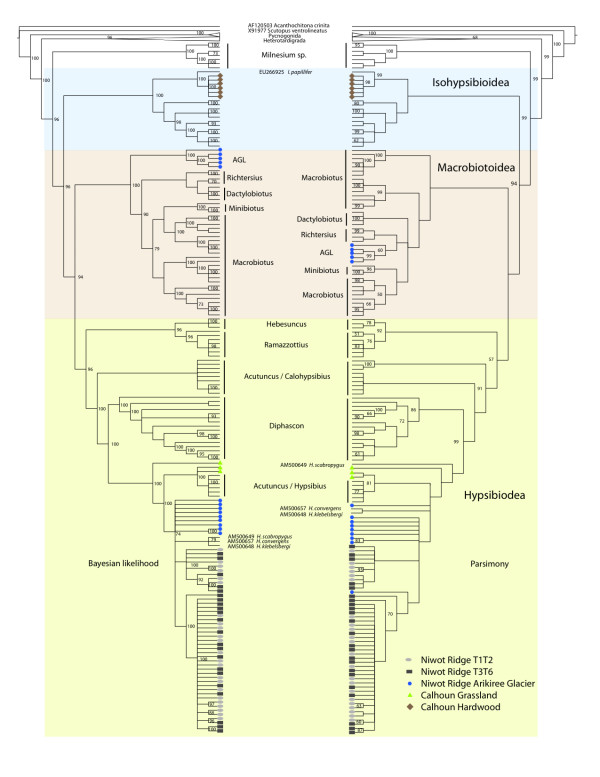
**Cladogram representations of phylogenetic trees obtained from TNT **[44]** and MrBayes **[45]** on tardigrades**. Bootstrap values below 50 and posterior probability values below 70 are not represented. All environmental sequences fall within the Eutardigrada.

Out of 1,814 nucleotide positions there were 900 variable sites, of which 677 were phylogenetically informative, comprising 68 unique phylotypes. Phylogenetic analysis clearly separates the two main groups of tardigrades: the Heterotardigrada and the Eutardigrada (Figure 3). Many of the environmental sequences from the high-elevation talus sites clustered into distinct clades, suggesting each clade may comprise a separate species. Eutardigrade sequences from soils near the Arikiree Glacier (AGL) grouped within the Macrobiotoidea and Hypsibiodea groups. Those within the Macrobiotidea are most closely related to *Richtersius coronifer*, a cosmopolitan species sampled from high elevation and arctic habitats [18]. The AGL sequences that grouped within the Hypsibiodea are related to those of the englacial dominating *Hypsibius *genus. These *Hypsibius *sequences from the AGL site are nearby and grouped with the two talus sites (T1T2 & T3T6).

The Calhoun Hardwood site sequences cluster closest to *Isohypsibius papillifer *typically found in Europe, Asia, Australia, & South America [19]. The genus *Isohypsibius *is composed of species that are widespread and has been documented circumglobally as well [19,20], (GBIF Sweden, 17 records; National Museum of Natural History, 10 records; Australian Antarctic Data Centre, 3 records).

The Calhoun Grassland sequences cluster basally with the Arikiree and Talus sites within the *Hypsibius *group, noted as "*Acutuncus*/*Hypsibius*" in contrast to another group labeled "*Acutuncus*/*Calohypsibius*" in Figure 3, (see [21,22] for clarification about taxonomic identification issues with *Hypsibius *and *Acutuncus*).

### Bdelloidea

**Figure 4:**
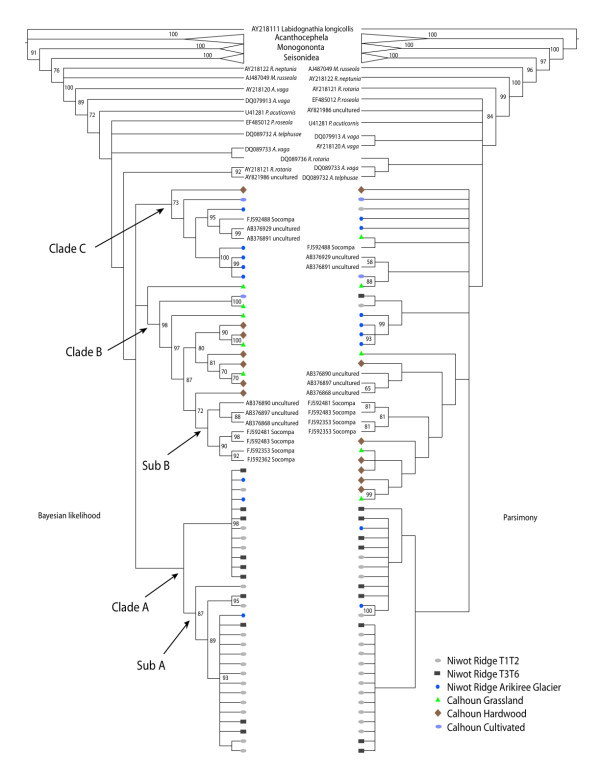
**Cladogram representations of phylogenetic trees obtained from TNT **[44]** and MrBayes **[45]** on bdelloid rotifers**. Bootstrap values below 50 and posterior probability values below 70 are not represented. All environmental sequences fall within the bdelloidea.

Out of 1638 sites 896 were variable and 718 where phylogenetically informative. The environmentally obtained sequences totaled 54 unique phylotypes (49 from this study). Phylogenetic analysis clearly separates all of the main clades of rotifers: Seisonidea, Monogononta and Bdelloidea (Figure 4). All of the environmental sequences we sampled grouped within the Bdelloidea. We also discovered three relatively diverse clades. The first is dominated by Niwot Ridge sequences (Clade A). One of the clades within Clade A (Sub A) is mainly dominated by sequence types from the T1T2 site. The second clade (Clade B) is dominated by those sequences from the Calhoun sites. What is interesting here is that the most derived cluster within Clade B contains uncultured sequences from Japan (Ibaraki upland soils) along with sequences from a high elevation site in Socompa, South America (Sub B) [23].

The final main group of sequences, Clade C, contains sequences from several locales, but mostly those from the AGL site. Again, like in Sub B, we observe uncultured sequence data from Japan (Fukushima and Ibaraki) clustering with a sequence from Socompa.

The lack of 18S rDNA sequence information in online data bases (14 bdelloid sequences in GenBank [24] as of this writing), makes the identification of environmentally obtained sequences even more difficult.

## Discussion

The development of clade-specific primers that allows characterization of eutardigrade and bdelloid rotifer communities from environmental samples should greatly increase our ability to discern the community diversity of these taxa in environmental samples. Moreover, the rDNA sequence data can be directly stored (within software packages like ARB [25]) and compared with other surveys that attempt to characterize invertebrate community composition [16,26].

We anticipate that environmental DNA surveys using clade-specific primers, like those we have developed, will be used to complement more directed studies that cultivate individual micro-eukaryotes as a means of more fully describing the diversity of ecological communities. We have yet to assess whether isolation of individuals and environmental DNA surveys yield different estimates of community composition, as is the case for surveys of bacteria (but see [16,26]) and bdelloid rotifers [27].

Environmental sequencing as shown here differs from other molecular survey methods [6,14,16,26] in that there is no need to pre-isolate the organisms of interest from soil (or other media), in order to amplify their DNA. Here, we simply extract total cellular DNA from all organisms in the soil and use targeted primers for the group of interest. This allows for a single DNA extraction prep instead of one DNA extraction prep for each targeted organism of interest.

### Eutardigrada

Although there are too few data to make robust biological inferences, several results are noteworthy. We found sequences from the highest elevation site in Colorado (near the Arikiree glacier) that grouped together with *R. coronifer*, a cosmopolitan morpho-species known to exist in high mountain and arctic habitats which is also known to survive extreme desiccation and temperatures down to -196°C [28]. Additionally, several sequences from the Calhoun hardwood forest were very similar to *Isohypsibius papillifer*, a widespread European species. Moreover, the genus *Isohypsibius *is ubiquitous, distributed from North America, Northern Europe, and Asia, all the way to Antarctica.

Interestingly, the sequences from the AGL site seem to have the most distant set of sequences compared to the other sites. One set of sequences is from within the Macrobiotidae, *Richtersius *group and the other from the Hypsibiodea, *Hypsibius *group. This is probably due to the longer duration of moist and wet soils that allows for a greater diversity of eutardigrade groups.

It is not too suprising that the majority of the Eutardigrade sequences amplified from the Talus and glacier sites are dominated by *Hypsibius*-related sequences. The Hypsibidae are known to dominate englacial habitats and are the dominate family of polar and cryoconite tardigrades. *Hypsibius *species are hydrophilic and are composed of bacteriophagous and/or algivorous feeding types. These biological factors aid in the colonization of nunatuks and glacial habitats (as reviewed in [29]).

However, several sequences from the Macrobiotidae were also found within the glacial habitat of the AGL site. Macrobiotidae are traditionally considered cosmopolitan occurring in many habitats, including those that are periodically frozen [29]. The AGL sequences cluster closest to the known sequences of *Richtersius sp. *(Figure 3). *Richtersius *have been the focus of many anhydrobiosis studies and have shown significant improvements in desiccation survival when many individuals aggregate together during anhydrobiosis [30]. This could lead to positive density dependence and even allow these animals to achieve greater monopolization [as reviewed in [31]] to local habitats that encounter extreme desiccation events like the high elevation AGL and talus sites. However, aggregation can create problems with environmental sequencing strategies like the one proposed here. If aggregation in the wild occurs within other eutardigrade groups then environmental sequencing may lead to amplification of only those extremely high-abundant clusters of animals.

### Bdelloidea

In contrast to the tardigrades, there was less agreement of support between the two different phylogenetic reconstruction methods of Bayesian and parsimony analysis for bdelloid rotifers. It was not possible to identify what bdelloids the environmental sequences were related to due to lack of abundant reference sequences. However, while it was possible to make some general statements about the bdelloid communities at the listed sample sites, the lack of resolution of 18S rDNA compared to 28S rDNA [32] makes it difficult to delineate the more recent clades of Bdelloidea (Figure 4). In fact, a similar level of poor resolution of bdelloids is also seen from phylogenies produced via cytochrome oxidase subunit 1 sequence data, wherein the early nodes are mostly saturated with polytomies (Robeson & Birky unpublished). Better resolution of this group at the tips of the phylogeny is often seen regardless of the phylogenetic reconstruction method chosen.

It is interesting that sequences from Socompa [23] cluster with the Calhoun sequences as opposed to other high elevation sites like the dry Talus, in Niwot Ridge. Although Socompa is a very high elevation site (5824 m above sea level), it is most likely similar in its microhabitat to the Calhoun sites, where there is greater moisture compared to the dry Talus. The Socompa site is characterized as a fumerole environment [23]. Typically fumaroles are areas where steam and volcanic gases vent out of the earth's crust due to the degassing of magma and/or geothermal heating of shallow ground water. This particular fumerole site is weakly active, creating an environment in which communities of mosses and liverworts are sustained by warm water vapor. The potentially similar microhabitats may be the reason for finding such similar sequence types in very different locales.

Bdelloid rotifers in particular show evidence for geographic structure among clades. Whether this apparent pattern reflects environmental filtering, priority effects (differences in arrival time that can have a lasting effect on differences in species dominance), or some other process remains to be seen. Nonetheless, the data presented here support the contention of [33], in which instances of endemism are seen (Clade A & B), with a few phylogenetic clusters of widespread bdelloids sampled from very different locales (Clade C and Sub B). It may be that harsher conditions in which there are very ephemeral moments of soil moisture creates higher levels of endemism of bdelloids, whereas environments in which soil moisture is sustained for longer periods of time allow for increased chances of long distance dispersal to suitable habitats and persistence. The location of the Socompa fumerole sites in the phylogeny (Figure 4) and its high similarity to sequences from Japan and within the Calhoun sites (Clade B & C) may be an indication of the latter point. One caveat here is that the 18s rDNA sequences are more conserved than their cytochrome oxidase subunit 1 counterparts [4,33] preserving more ancient than contemporary relatedness.

## Conclusion

Large-scale surveys of rotifer and tardigrade diversity using traditional approaches makes for a large and unwieldy set of tasks (i.e. difficulties associated with isolation, identification and enumeration of organisms that do not preserve any discernable morphological characters).

Environmental sequencing is valuable for performing large-scale surveys of the diversity of organisms that cannot be cultured or grown in the laboratory or in which species are difficult to distinguish using phenotypic characters. The DNA sequences obtained from non-cultured based methods can be identified post-hoc (placed phylogentically) as closely related sequences are obtained from morphologically identified conspecifics. Our environmental sequence based approach, which does not require culturing or isolation of animals from soils, provides a rapid and large-scale screening for the presence, absence and diversity of Bdelloidea and Eutardigrada in a variety of soils.

We have shown that targeted amplification of eutardigrades and bdelloid rotifers are possible from a range of soil types. This sequence data can be used to quickly assess the peculiar biogeography [31,34] and genetic diversity of soil samples, more often informing us of dominate groups within each sample.

It should also be emphasized that environmental sequencing strategies like this are not intended to replace, but instead complement ongoing morphological work, explore the possible effects of heterogeneity within individuals, and the effect of this variation on phylogenetic analysis [35]. This highlights the need for morphological taxonomists and molecular ecologists to work together in order to make environmental sequencing methods, like the one proposed here, more robust. In particular, studies such as these are most empowered by the cataloging of sequence data from vouchered specimens.

## Methods

### Soil DNA extraction

Soil samples (~5 g) were taken from all sites. Three sites from within the Niwot Ridge Long Term Ecological Research (LTER) area in the Front Range of the Colorado Rocky Mountains, United States of America (40° 03' N, 105° 35' W). These sites are: the Arikiree Glacier (AGL), Talus site 1 (T1T2), and Talus site 2 (T3T6) as described previously by [36]. Other soil samples were also obtained from the Calhoun Experimental Forest (managed by the US Department of Agriculture located in northwestern South Carolina in the Piedmont region, 34.5°N, 82°W), these sites are: Hardwood (H), Grassland (G), and Cultivated (C). Total cellular DNA was extracted from soil using the PowerSoil DNA Isolation Kit #12888 (Mo Bio Laboratories, Inc, Carlsbad, CA).

### Primer development

Only forward 18S SSU primers were developed to target specific groups (bdelloids and eutardigrades). Primer development entailed downloading all available target sequences of interest along with their closest set of outgroup taxa from GenBank [24] and aligned using Muscle [37] and edited in ARB [25] to align conserved regions only. A region of bases unique to the target group that excluded as many matches as possible to the outgroup taxa were chosen for primer development. Bdel_2: 5'-CGG CTC ATT ACA TCA GCT ATA ACT T-3' was used for bdelloid rotifers, and Tard_1: 5'-TCT CAG TAC TTG CTT TAA CAA GGC-3' was used for eutardigrades. Amplicon products produced were ~1700 base pairs in length. All eutardigrade and bdelloid rotifer environmental sequences had a sequence identity to those in GenBank ranging from 91 to 98% with a query coverage of 99 to 100% and 95-99% with a query coverage of 97-100% respectively.

Other 'universal' primers used in this study were taken or derived from [38-40] and are listed here as follows: 18S2a: 5'-GAT CCT TCC GCA GGT TCA CC-3'; 18S3: 5'-GAC TCA ACA CGG GAA ACC TCA CC-3'; 18S10: 5'-CTA AGG GCA TCA CAG ACC-3'

### PCR

The reverse primer 18S2a was used in conjunction with either the Tard_1 or Bdel_2 primer in order to amplify the DNA of either eutardigrades or bdelloid rotifers directly from soil. The PCR cycling conditions were as follows: initial denaturation at 94°C for 2 min, followed by 40 cycles of: 94°C for 30", 60°C for 30", 72°C for 2', with a final extension at 72°C for 10'. PCR reaction contained (all reagents from Invitrogen, Carlsbad, CA, USA) 1× PCR Buffer, 1.5 mM MgCl_2_, 0.2 μM dNTPs, 0.4 μM of each primer, *Taq *polymerase (0.5 units), template DNA: 2 μL.

### Cloning & Sequencing

The final PCR product was purified using the Wizard SV Gel and PCR Clean-up System (Promega, Madison, WI) or the QIAquick Gel Extraction Kit 28704 (QIAGEN, Valencia, CA). Purified PCR product was then cloned using the Invitrogen TOPO TA Kit (with pCR2.1-TOPO vector) with One Shot TOP10 Chemically Competent E. coli (K4500-01). Pelleted cells were sent to Functional Biosciences, Inc (Madison, WI) for sequencing. The 18S3 and 18S10 primers were only used at this step for internal sequencing along with M13 primers to generate robust sequence data for contig assembly.

### Sequence analysis

Sequence data was assembled, vector and primer sequence removed, then edited by hand using Sequencher 4.7 (Gene Codes Cooporation, Ann Arbor MI). Sequences where chimera-checked using the Bellerophon server [41] and determined that no chimeras by sample site amplicons were detected. Usable data were then exported for BLAST [42] searches. All sequences produced and/or used in this study are listed by accession in Table 1.

**Table 1:** List of Accession numbers by major groups. Sequences used as guides as well as those generated from this study.

**Environmentally obtained Bdelloids (this study)**	GQ922286 - GQ922334
**Bdelloidea**	AJ487049, AY21812-AY218122, DQ079913, DQ089732, DQ089733, DQ089736, EF485012, U41281

**Uncultured Bdelloidea**	AB376868, AB376890, AB376891, AB376897, AB376929, AY821986, FJ592353, FJ592362, FJ592481, FJ592483, FJ592488

**Acanthocephela**	AF001841, AY218124, AY423346, AY423347, AY830151, AY830156, EF107645, EF107648

**Monogononta**	AF001840, AF092434, AY218117, AY218119, DQ297692, DQ297698, DQ297723

**Seisonidea**	AF469411, DQ089737, DQ297761

**Gnathostomulida**	AY218111

**Environmentally obtained Eutardigrades (this study)**	GQ922218 - GQ922285

**Eutardigrada**	AF056023, AM500646-AM500649, AM500651, AM500652, AY582120-AY582123, DQ839601-DQ839605, EF620401-EF620404, EF632424-EF632432, EF632436, EF632437, EF632439, EF632441, EF632443-EF632445, EF632447, EF632449, EF632452, EF632467, EF632468, EF632471, EF632473, EF632475, EF632477, EF632479, EF632485, EF632488, EF632490, EF632493, EF632494, EF632497, EF632503, EF632509, EF632511, EF632513, EF632515, EU038077-EU038081, EU266923-EU266937, EU266939-EU266955, EU266957-EU266959, U32393, U49909, U49912, X81442, Z93337

**Heterotardigrada**	AY582118, AY582119, DQ839606, DQ839607, EF632433, EF632453, EF632456, EF632466, EU266960, EU266961, EU266962, EU266963, EU266964, EU266965, EU266966, EU266967, EU266968, EU266969, EU266970, EU266973, EU266975

**Pycnogonida**	AF005438, AF005441

**Mollusca**	AF120503, X91977

Pre-aligned guide and outgroup sequences were downloaded from the SILVA database [43]. The SILVA aligner was used to align the environmental 18s rDNA SSU sequence data according to secondary structure [43]. The data was further edited by eye and exported from ARB [25] using an 'in-house' filter to remove highly ambiguous regions of the alignment. All terminal gaps in the alignment were converted to missing (i.e. as '?' characters) and gaps '-' counted as a 5^th ^character state. TNT [44] and a multi-core version of MrBayes [45] were used to confirm the phylogenetic placement of environmentally obtained sequences. Parsimony analysis was performed by generating 1000 bootstrap replicates. Before re-sampling, the trees were collapsed using TBR. Each bootstrap replicate was composed of twenty iterations of 'Wagner addition trees' (trees formed by sequentially adding the taxa at the best available position, using Fitch parsimony) followed by swapping with TBR, the single best tree was then used for random sector searches and trees saved. MrBayes was used to perform 5 and 8 million generations using the GTR + G + I model of evolution as specified by MultiPhyl Online on the bdelloid and eutardigrade data sets respectively [46].

## Authors' contributions

MSR conceived of and directed the project as well as developed the clade-specific primers. MSR, KRF, JW, & BA sampled, extracted and/or amplified and sequenced DNA from several sites or individual organisms. MSR, EKC, APM & SKS participated in the design and coordination of the study. APM, SKS, & BA guided and provided suggestions throughout the project and aided in the interpretation of the data. All authors helped to draft the manuscript. All authors read and approved the final manuscript
